# Phorbol myristate acetate suppresses breast cancer cell growth via down-regulation of P-Rex1 expression

**DOI:** 10.1007/s13238-016-0261-x

**Published:** 2016-03-28

**Authors:** Chuu-Yun A. Wong, Haihong Jiang, Peter W. Abel, Margaret A. Scofield, Yan Xie, Taotao Wei, Yaping Tu

**Affiliations:** Department of Pharmacology, Creighton University School of Medicine, Omaha, NE 68178 USA; National Laboratory of Biomacromolecules, Institute of Biophysics, Chinese Academy of Sciences, Beijing, 100101 China

**Dear Editor,**

P-Rex1 is a Rac-selective guanine nucleotide exchange factor (GEF) that is synergistically activated by G-protein coupled receptors and receptor tyrosine kinases (Welch et al., 2002). We previously reported that aberrantly upregulated P-Rex1 promotes prostate cancer metastasis by activating Rac1 signals (Qin et al., 2009). P-Rex1 is also highly overexpressed in estrogen receptor-positive and ErbB2-overexpressing human luminal breast tumors, which correlates with the aggressiveness of human breast cancer and poor outcome in breast cancer patients (Montero et al., 2011; Sosa et al., 2010). Silence of endogenous P-Rex1 blocks breast cancer cell proliferation, tumorigenesis, and motility (Montero et al., 2011; Sosa et al., 2010). Therefore, P-Rex1 is an important mediator in cancer progression and could be a potential therapeutic target.

Protein kinase C (PKC), a family of serine-threonine kinases, has been implicated in breast cancer progression (Urtreger et al., 2012). PKC isozymes are classified into conventional (α, β, and γ), novel (δ, ε, η, and θ), and atypical (ζ and λ) PKCs. Expression profiles of PKC isoforms vary among different breast cancer cell lines (Urtreger et al., 2012). Phorbol 12-myristate 13-acetate (PMA), a structural homolog of diacylglycerol (DAG), activates conventional and novel PKCs. PMA treatment induces breast cancer cell growth arrest via sustained up-regulation of the cell-cycle inhibitor p21 (WAF1/CIP1) (Barboule et al., 1999; Fortino et al., 2008). Interestingly, Rac1 was reported to be overexpressed or hyperactive in breast cancer tissues (Schnelzer et al., 2000) and hyperactivity of Rac1 suppressed p21 (WAF1/CIP1) expression in cancer cells (Knight-Krajewski et al., 2004). Since P-Rex1 functions as a Rac1 activator in cancer cells (Qin et al., 2009; Sosa et al., 2010), the purpose of the present study was to determine the role of P-Rex1 in PMA inhibition of breast cancer cell growth.

Both MCF-7 and BT-474 cell lines, derived from human luminal breast cancers, are ER-positive and highly express P-Rex1 (Sosa et al., 2010). MCF-7 cells are also ErbB2-positive whereas BT-474 cells are ErbB2-overexpressed. Thus, these two cell lines were chosen for our studies. Western blot analysis showed that the P-Rex1 protein expression level in BT-474 cells is 4.5-fold higher than that in MCF-7 cells (Fig. 1A). Thirty hours treatment with PMA caused a concentration-dependent decrease in P-Rex1 protein levels in both MCF-7 and BT-474 cells with a maximum reduction of 87.2% ± 1.1% and 57.0% ± 8.6 %, respectively, at a concentration of 10 ng/mL PMA (Fig. 1B). PMA also significantly attenuated growth of both MCF-7 and BT-474 cells in a concentration-dependent manner with an inhibition of 77.8% ± 12.4% and 50.6% ± 3.7%, respectively, at 10 ng/mL PMA (Fig. 1C). Interestingly, PMA-induced inhibition of cell growth is correlated to the degree of P-Rex1 down-regulation in MCF-7 and BT-474 cells. Thus, a recovery assay was performed to determine whether PMA inhibition of breast cancer cell growth is P-Rex1 dependent. As shown in Fig. 1D inset, expression of recombinant P-Rex1 restored the P-Rex1 expression level in PMA-treated MCF-7 cells. PMA treatment dramatically reduced the growth of control MCF-7 cells but not cells transfected with P-Rex1. Expression of recombinant P-Rex1 had little effect on MCF-7 cell growth in the absence of PMA but completely restored cell growth in the presence of PMA (Fig. 1D). Although transfection of recombinant P-Rex1 plasmid only slightly increased P-Rex1 protein level in untreated BT-474 cells, it still partially restored the P-Rex1 protein expression in PMA-treated BT-474 cells (Fig. 1E, inset). More importantly, expression of recombinant P-Rex1 increased PMA-treated BT-474 cell growth by 1.7-fold, which equals 70% of untreated control cells (Fig. 1E).

**Figure 1:**
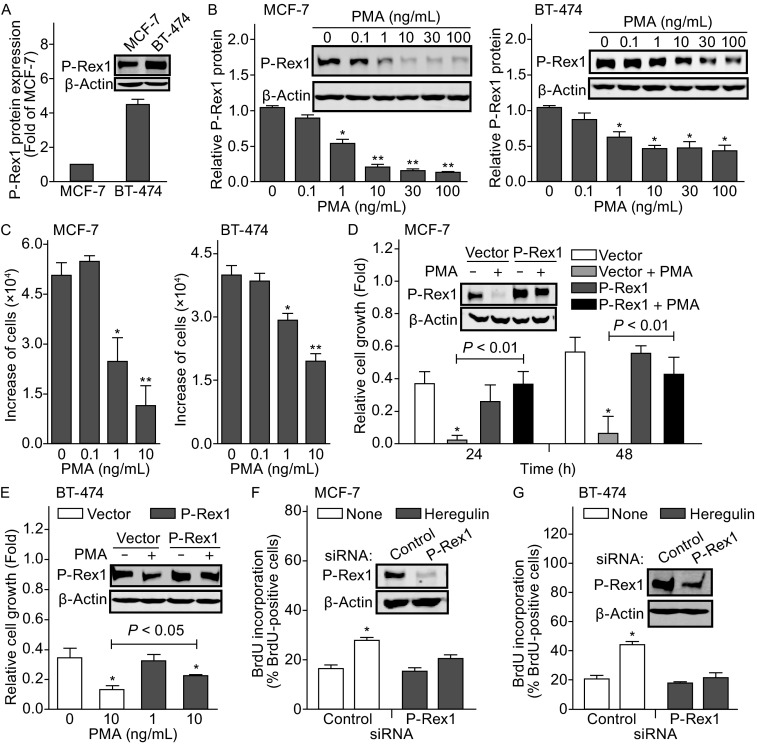
**PMA suppresses breast cancer cell growth through P-Rex1 down-regulation**. (A) Western blot analysis of P-Rex1 protein expression in MCF-7 and BT-474 cells. Data shown are means ± SEM (*n* = 3). (B) PMA concentration-dependent down-regulation of P-Rex1 protein expression in MCF-7 and BT-474 cells. Data are means ± SEM (*n* = 3) with *P < 0.01 and **P < 0.001 compared with untreated cells, normalized by β-actin. (C) PMA concentration-dependent suppression of MCF-7 and BT-474 cell growth. Cells were cultured in the presence or absence of PMA for 48 h (MCF-7) or 72 h (BT-474). Data shown are means ± SEM (*n* = 5 of duplicates) with *P < 0.05 and **P < 0.001 as compared with untreated cells. (D and E) Expression of recombinant P-Rex1 blocked PMA inhibition of MCF-7 and BT-474 cell growth. Cells transfected with control vector or P-Rex1 were cultured in the absence or presence of PMA (10 ng/mL) for 24 h and 48 h (MCF-7) or 48 h (BT-474). Relative cell growth refers to increased cell number normalized by cell number prior to PMA treatment. Data are means ± SEM (*n* = 5 of duplicates) with *P < 0.01 as compared to cells without PMA treatment. (F and G) Silence of endogenous P-Rex1 by its siRNA abolished heregulin (100 ng/mL)-stimulated MCF-7 and BT-474 cell proliferation, determined by BrdU incorporation assay. Data are means ± SEM (*n* = 3 of triplicates) with *P < 0.01. Insets: Western blot analysis of P-Rex1 and β-actin protein expression in MCF-7 and BT-474 cells

Hyperactived ErbB receptor signaling has been frequently characterized in breast carcinomas (Hynes and Lane, 2005). P-Rex1 is an essential mediator of ErbB signaling in breast cancer (Sosa et al., 2010). Thus, we silenced endogenous P-Rex1 expression in MCF-7 and BT-474 cells by over 80% using P-Rex1 specific siRNA (Fig. 1F and 1G, inset). Treatment with heregulin (100 ng/mL), an ErbB activating ligand, increased proliferation of MCF-7 (Fig. 1F) and BT-474 (Fig. 1G) cells transfected with control siRNA by 1.8-fold and 2.1-fold respectively, as indicated by BrdU incorporation assay. However, this stimulatory effect was significantly reduced in cells transfected with P-Rex1 siRNA (Fig. 1F and 1G). Together, our data suggest that P-Rex1 functions as a key molecule in breast cancer cell growth and that down-regulation of P-Rex1 contributes to PMA suppression of MCF-7 and BT-474 cell growth.

PMA mimics DAG in the cellular membrane to activate PKC. Treatment with PMA (10 ng/mL) significantly increased PKC kinase activity in both MCF-7 and BT-474 cells, which was completely blocked by pre-treatment with the general PKC inhibitor Gö6983 (2 µmol/L) (Fig. S1A). Western blot assay showed that both MCF-7 and BT-474 cells express PMA-sensitive conventional PKC isoforms (α and β) and novel PKC isoforms (δ, ε and η). MCF-7 cells also express the novel PKC isoform (θ). In addition, PMA-insensitive atypical PKC isoforms (ι and λ) were also detected in both MCF-7 and BT-474 cells (Fig. S1B). Immunofluorescence staining analysis showed that 30 min after treatment of MCF-7 cells with PMA (10 ng/mL), PKCα appeared to translocate to the cell plasma membrane and was enriched in the nucleus whereas PKCε mainly translocated to the nucleus (Fig. S1C). These effects further indicate PKC activation upon stimulation with PMA. Interestingly, pre-treatment with Gö6983 (2 µmol/L) reduced the PMA inhibitory effect on P-Rex1 protein expression from 85.6% ± 2.6% to 33.6% ± 7.7% in MCF7 cells (Fig. 2A, left) and completely blocked PMA-induced down-regulation of P-Rex1 protein in BT-474 cells (Fig. 2A, right). In contrast, 100 nmol/L of Gö6976, a selective inhibitor of conventional PKC isoforms, had no effect on PMA down-regulation of P-Rex1 expression in MCF-7 and BT-474 cells (Fig. 2A). As expected, Gö6983 but not Gö6976 blocked PMA-induced suppression of MCF-7 and BT-474 cell growth (Fig. 2B). Thus, activation of novel PKC isoforms but not conventional PKC isoforms may be involved in PMA-induced P-Rex1 down-regulation and suppression of breast cancer cell growth.

**Figure 2:**
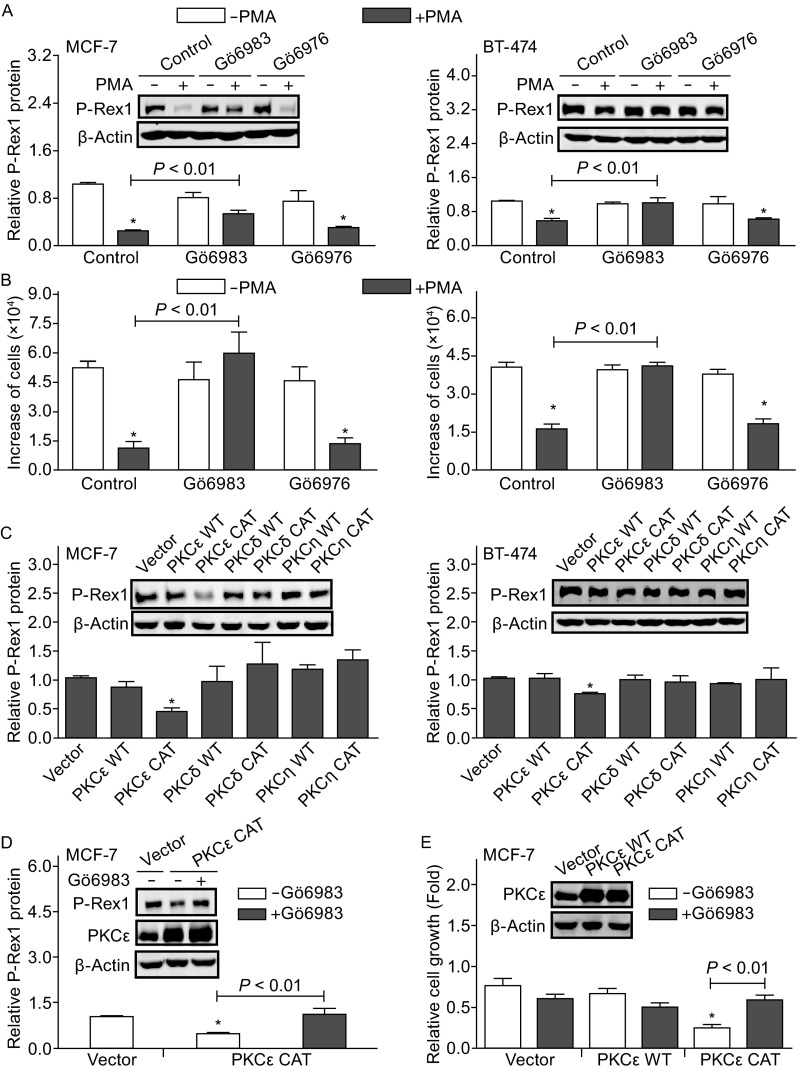
**PKCε activation contributes to PMA suppression of breast cancer cell growth through P-Rex1 down-regulation**. The general PKC inhibitor Gö6983 (2 µmol/L), but not the conventional PKC isoform inhibitor Gö6976 (100 nmol/L), attenuated PMA (10 ng/mL) suppression of P-Rex1 expression (A) and breast cancer cell growth (B). (A) Western blot analysis of P-Rex1 expression. Data are means ± SEM (*n* = 3) with *P < 0.01 compared to cells without PMA treatment. (B) Cell growth assay. Cells were cultured in the presence or absence of PMA for 48 h (MCF-7) or 72 h (BT-474). Data are means ± SEM (*n* = 5 of duplicates) with *P < 0.01 compared to cells without PMA treatment. (C) Expression of PKCε constitutively active form (CAT), but not PKCε wild-type (WT), PKCδ or PKCη WT and CAT mutant, down-regulated P-Rex1 expression in MCF-7 and BT-474 cells. Data are means ± SEM (*n* = 3) with *P < 0.01 compared to cells transfected with vector. (D) Gö6983 (2 µmol/L) attenuated PKCε CAT-induced down-regulation of P-Rex1 expression in MCF-7 cells. (E) Expression of PKCε CAT, but not its WT, suppressed MCF-7 cell growth, which is blocked by treatment with Gö6983 (2 µmol/L). Data are means ± SEM (*n* = 3 of duplicates) with *P < 0.01 compared to cells transfected with vector. Insets: Representative Western blot images of P-Rex1, PKCε, and β-actin protein expression in breast cancer cells

It should be noted that Gö6983 is a PKC-selective but not specific inhibitor. At the concentration used (2 µmol/L) it also inhibits many other kinases. Our data does not exclude the possibility of involvement of other kinases in PMA effects in breast cancer cells. Thus, we investigated whether expression of active novel PKC isoforms (PKCε, PKCδ, and PKCη) directly modulate P-Rex1 expression and breast cancer cell growth. As shown in Fig. 2C, ectopic expression of wild-type (WT) or constitutively active (CAT) PKCδ or PKCη had no significant effect whereas expression of PKCε CAT mutant down-regulated P-Rex1 protein expression in MCF-7 and BT-474 cells by 40%–60% (Fig. 2C). In contrast, expression of PKCε WT had no significant effect on P-Rex1 expression (Fig. 2C), suggesting that PKCε overexpression by itself is not sufficient to induce P-Rex1 down-regulation in breast cancer cells. To further investigate whether PKCε activity is essential for P-Rex1 down-regulation, Gö6983 was used to inhibit the constitutively active PKCε activity. As shown in Fig. 2D, Gö6983 completely abolished PKCε CAT-induced P-Rex1 down-regulation. Interestingly, expression of PKCε CAT mutant but not WT suppressed MCF-7 cell proliferation by 70%, which was also attenuated by Gö6983 (Fig. 2E). Together, our data suggest that activation of PKCε is more important than overexpression of PKCε in regulating P-Rex1 expression and breast cancer cell growth.

In summary, our study is the first to use a small molecule, PMA, to target P-Rex1 expression levels to suppress breast cancer cell growth. PMA itself has both oncogenic and anti-tumorigenic properties by direct or indirect modulation of various cellular targets (Griner and Kazanietz, 2007). Previous studies suggest that induction of the cell-cycle inhibitor p21 (WAF1/CIP1) is involved in connecting the PMA-activated PKC signaling pathways to the breast cancer cell cycle regulatory machinery, leading to cell growth arrest (Barboule et al., 1999; Fortino et al., 2008). Interestingly, Rac1 was reported to be overexpressed or hyperactive in breast cancer tissues and hyperactivity of Rac1 suppressed p21 (WAF1/CIP1) expression in cancer cells. Since P-Rex1 functions as a Rac1 activator in cancer cells, PMA-down-regulation of P-Rex1 expression should result in reduction of Rac1 activity, leading to increased expression of p21 (WAF1/CIP1). Thus, our study provides a potential molecular mechanism underlying PMA suppression of breast cancer cell growth.

Our studies further showed that active PKCε, but not the other PMA-sensitive PKC isoforms, down-regulates P-Rex1 expression and suppresses breast cancer cell growth. PKCε has a unique role in regulating cell-signaling pathways in cancer (Griner et al., 2007; Urtreger et al., 2012). Elevated PKCε levels were correlated with breast cancer aggressiveness (Pan et al., 2005). Our study presents the first evidence suggesting that the PKCε/P-Rex1 pathway may be an attractive new target for therapeutic intervention, which provides an additional approach for improving the current treatment of breast cancer. For example, estrogen receptor-targeted therapies have significantly reduced breast cancer mortality. However, resistance generally emerges because various growth factor receptors such as ErbB2 can transactivate estrogen receptors in an estrogen-independent manner, contributing to tumor growth. Trastuzumab, a monoclonal ErbB2 antibody, has significant clinical benefit for patients with ErbB2-elevated breast tumors (Smith et al., 2007). However, patients may also develop resistance within 1 year of treatment. A common feature of the possible mechanisms of resistance is Rac1 activation and inactivation of Rac1 reduces Trastuzumab resistance in breast cancer cells (Zhao et al., 2011). P-Rex1 is highly expressed in human breast cancers with high ErbB2 and estrogen receptor expression and functions as a Rac-specific activator at a convergence point downstream of ErbB receptors and other growth factor receptors (Montero et al., 2011; 2013; Sosa et al., 2010). Thus, it has been suggested as an attractive therapeutic target (Sosa et al., 2010). Understanding PKCε-dependent P-Rex1 down-regulation may provide a novel strategy for development of chemotherapeutic agents for P-Rex1-overexpressing breast cancer patients that develop resistance to anti-estrogen and/or anti-ErbB2 therapies.

## Electronic supplementary material

Below is the link to the electronic supplementary material.

Supplementary material 1 (PDF 609 kb)
