# Supplementary material for: Phorbol myristate acetate suppresses breast cancer cell growth via down-regulation of P-Rex1 expression
Source: Protein Cell. 2016 Mar 28;7(6):445–9. doi: 10.1007/s13238-016-0261-x (PMC4887325; doi:10.1007/s13238-016-0261-x)
Supplement: Supplementary file 1 — Supplementary material 1 (PDF 609 kb) [file 13238_2016_261_MOESM1_ESM.pdf]

Supplementary figure 1

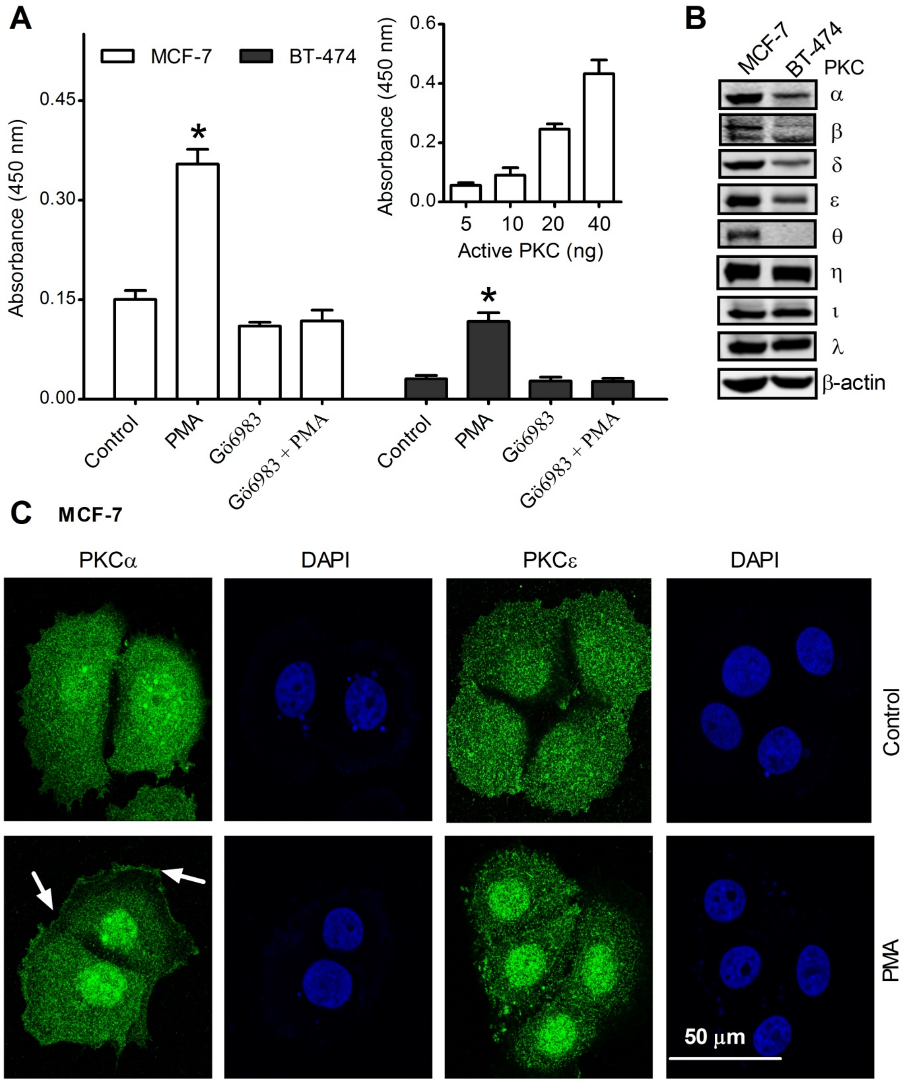

Fig. S1. PMA treatment induces PKC activation in MCF-7 and BT-474 cells. (A) The general PKC inhibitor Gö6983 (2 μM) blocked PMA (10 ng/ml)-stimulated PKC kinase activity. The PKC kinase activity was measured at an absorbance of 450 nm. Data are means ± S.E. (n = 3 of duplicates) with *$p < 0.01$ compared to control or Gö6983 treated cells. Inset: PKC kinase activity of varying quantities of purified active PKC. (B) Western blot analysis of expression of PKC isoforms in MCF-7 and BT-474 cells. Images shown are representative of three separate experiments. (C) Immunofluorescent detection of translocation of PKCα and PKCε induced by 30 min treatment with 10 ng/ml PMA. Images are representative fields from at least 3 independent experiments. Arrows point to plasma membrane.

Supplementary materials and methods

Cell lines, plasmid, and reagents

MCF-7 and BT-474 cells were from the American Type Culture Collection (Manssas, VA). MCF-7 cells were maintained in IMEM medium (Corning Cellgro) (Manassas, VA) and 10% fetal bovine serum (Atlanta Biologicals) (Lawrenceville, GA) with 10 µg/ml insulin (Cell Applications, Inc.) (San Diego, CA), 1x non-essential amino acids (Invitrogen) (Grand Island, NY), and 10 mM HEPES (pH7.4) (MP Biomedicals) (Solon, OH). BT-474 cells were cultured in DMEM medium (Thermo Fisher Scientific) (Logan, UT) with 10% fetal bovine serum.

The constitutively active (CAT) PKCε (A159E) mutant was generated from PKCε wild-type (WT) with a Stratagene QuikChange mutagenesis kit (Schönwasser *et al.*, 1998). pcDNA3 plasmids encoding PKCε WT, PKCδ WT and its CAT mutant, PKCη WT and its CAT mutant were from Addgene (Cambridge, MA). P-Rex1 antibody was from Sigma-Aldrich (St. Louis, MO). PKCε and PKCη antibodies were from Millipore (Billerica, MA) and GenScript (Piscataway, NJ). β-actin antibody was from Santa Cruz Biotechnology (Santa Cruz, CA). PKC antibody sampler kit was from BD Biosciences (San Jose, CA). 3-[1-[3-(dimethylamino)propyl]-5-methoxy-1H-indol-3-yl]-4-(1H-indol-3-yl)-1H-pyrrole-2,5-dione (Gö6983) was from Tocris Bioscience (Bristol, United Kingdom). 5,6,7,13-Tetrahydro-13-methyl-5-oxo-12H-indolo[2,3-a]pyrrolo[3,4-c]carbazole-12-propanenitrile (Gö6976), PMA and human heregulin were from Millipore. Other reagents were from either Sigma-Aldrich or Thermo Fisher Scientific.

Western blot (WB)

Protein samples extracted from exponentially growing cells were loaded on SDS-PAGE, electrophoresed and transferred to Immobilon-FL membrane (Millipore) as described previously (Wong *et al.*, 2011). Primary antibodies were used to identify the relevant protein of interest and the loading control (β-actin). IRdye700 or IRdye800 labeled secondary antibodies (LI-COR Biosciences) (Lincoln, NE) were used for protein band detection. The images were captured with a LI-COR Odyssey infrared imaging system (LI-COR Biosciences). For the quantification of the WB results, the intensity of each band was divided by the β-actin intensity of that same sample and subsequently divided by the corrected intensity of the untreated sample of the same blot.

Expression of recombinant PKC isoforms or P-Rex1

MCF-7 or BT-474 cells (1 x 10^6) were electroporated with 1.5 μg of either pcDNA3.1 control vector or vector encoding PKCε WT, PKCε CAT, PKCδ WT, PKCδ CAT, PKCη WT, or PKCη CAT. Cells were seeded on 6-well plates and harvested 72 h later for WB analysis of P-Rex1 expression. To determine the effect of exogenous P-Rex1 on PMA inhibition of breast cancer cell growth, MCF-7 and BT-474 cells were transfected with control vector or vector encoding P-Rex1 48 h prior to PMA (10 ng/ml) treatment and were counted 24 and/or 48 h later.

Cell growth assay

MCF-7 cells transfected with PKC isoforms for 24 h were seeded into 24-well plates at a density of 5×10^4 cells/well in IMEM phenol red-free medium supplemented with 2% fetal bovine serum. Cells were cultured in the presence or absence of 2 μM of the PKC inhibitor Gö6983 for 48 h. Cell number was counted with a hemocytometer (Fisher Scientific). Relative cell growth refers to increased cell number, normalized by cell number before Gö6983 treatment.

Silence of endogenous P-Rex1 by siRNA and 5-bromo-2'-deoxyuridine (BrdU) cell proliferation assay

MCF-7 and BT-474 cells (1×10^6) were electroporated with 300 nM of control siRNA (negative control #1 siRNA, Ambion (Austin, TX) or synthesized P-Rex1 siRNA (target sequence of human P-Rex gene: 5′-GCAACGACTTCAAGCTGGTGGAGAA-3′) using Nucleofector kits with an Amaxa Nucleofector System (Lonza) (Allendale, NJ) and cells were cultured for 48 h. Cells were then re-seeded on coverglasses in 12-well plates (1 x 10^5cells/well) and cultured in serum-free medium without or with heregulin (100 ng/ml) for 60 h. 10 μM BrdU from BD Pharmigen (San Jose, CA) was added for 18 h at 37°C. The cells were then incubated with anti-BrdU antibody (1:400) from Cell Signaling Technology (Danvers, MA) for 2 h at room temperature, followed by incubation with fluorescein isothiocyanate-conjugated secondary antibody (Sigma-Aldrich). The coverslips were then mounted on slides using Vectashield Mounting Medium with 4',6-diamidino-2-phenylindole (DAPI). Microscopy images were obtained using a Nikon Eclipse 80i microscope and Cool-SNAP CF camera (Nikon) (Tokyo, Japan). BrdU incorporation was calculated based on the number of BrdU positive cells divided by total DAPI stained cells. At least 100 cells were counted in each individual experiment.

Non-radioactive measurement of PKC kinase activity

MCF-7 and BT-474 cells were cultured overnight in IMEM medium supplemented with 2% fetal bovine serum and then treated without or with PMA (10 ng/ml) for 60 min. Cells were lysed on ice for 30 min with the lysis buffer (20 mM MOPS, pH 7.4, 150 mM NaCl, 5 mM EGTA, 2 mM EDTA, 1% NP40, 1 mM phenylmethylsulfonyl fluoride, 1 mM sodium orthovanadate, 1 mM dithiothreitol, 1 mM benzamidine and 10 μg/ml of leupeptin and aprotinin). Following sonication (3 × 10 sec pulses), the cell lysates were centrifuged at 13,000 ×g for 15 min and the supernatants were collected for PKC kinase activity assay.

50 μg of cell lysates were loaded in duplicate wells on the PKC kinase activity plate of a PKC kinase activity assay kit (Enzo Life Sciences, Farmingdale, NY). The assay was performed following the manufacturer's instruction. The PKC kinase activity was measured at an absorbance of 450 nm on the Synergy™ HTX Multi-Mode Microplate Reader from BioTek (Winooski, VT). Varying quantities of purified active PKC were also assayed to generate a standard curve.

Immunofluorescence microscopy

Monolayered MCF-7 cells on coverglass slides were cultured overnight in IMEM medium supplemented with 2% fetal bovine serum. Cells were treated with PMA (10 ng/ml) for 30 min and then fixed with 4% paraformaldehyde for 10 min. PKCα and ε isoforms were visualized by anti-PKCα poly-clonal and anti-PKCε mono-clonal antibodies (BD bioscience), followed by Alexa Fluor 488-labeled anti-rabbit IgG or anti-mouse IgG, respectively. Images were captured by using a confocal microscope (Leica TCS SP8 MP) and LAS imaging software.

Statistical analysis

All experiments were repeated 3-5 times in duplicates or triplicates. Results are expressed as the mean ± S.E.. Groups were compared using a Student's t-test for unpaired observations or a 2-way ANOVA with the Bonferroni correction where there were multiple comparisons. A p value <0.05 was considered to be significant.

Supplementary references:

Schönwasser DC, Marais RM, Marshall CJ, Parker PJ (1998) Activation of the mitogen-activated protein kinase/extracellular signal-regulated kinase pathway by conventional, novel, and atypical protein kinase C isotypes. Mol Cell Biol 18:790-798.

Wong CY, Wuriyanghan H, Xie Y, Lin MF, Abel PW, Tu Y (2011) Epigenetic regulation of Phosphatidylinositol 3,4,5-Triphosphate-dependent Rac exchanger 1 gene expression in prostate cancer cells. J Biol Chem 286:25813-25822.
